# The Serious Challenge of Occult Hepatitis B Virus Infection-Related Hepatocellular Carcinoma in China

**DOI:** 10.3389/fmicb.2022.840825

**Published:** 2022-02-07

**Authors:** Renxiang Xia, Jing Peng, Jian He, Ping Jiang, Chunyan Yuan, Xiaoli Liu, Yunqing Yao

**Affiliations:** Department of Infectious Diseases, The First Affiliated Hospital of Chongqing Medical University, Chongqing, China

**Keywords:** hepatocellular carcinoma, HbsAg(-), hepatitis B virus, occult HBV infection, HBcAb(+)

## Abstract

**Background:**

It is unknown how many people in China have chronic occult hepatitis B virus (HBV) infection (OBI) [chronic HBV infection with negative serum hepatitis B surface antigen (HBsAg) (N-HBsAg)]. Their clinical and virological characteristics, especially the correlation between the OBI and hepatocellular carcinoma (HCC), are still elusive and need to be investigated, including prevention, early diagnosis, and treatment strategies.

**Methods:**

138 patients with HCC related to OBI were screened from 698 patients of HCC associated with HBV infection, their characteristics of epidemiology, clinical, biochemistry, virology, diagnostics, and therapeutics were analyzed retrospectively. Furthermore, the correlation between virological features and clinical features was investigated.

**Results:**

It was found that 19.8% (138/698) of patients with HBV-related HCC were OBI, of which 79.7% (110/138) were men, and 20.3% (28/138) were women. Most of the patients with OBI-related HCC were older men, and the median age was 63.2 years. In total 78.3% (108/138) of the patients had apparent right upper abdomen discomfort and/or pain and then sought medical examination, while 21.7% (30/138) of the patients were identified by health examination. A total of 10.9% (15/138) of the patients were admitted with chronic infection of HBV, and 2.2% (3/138) of the patients were admitted with a family history of hepatitis B. The alpha-fetoprotein (AFP) serum-positive rate was 39.1% (54/138). Tumor lesions >5.0 cm, with intrahepatic and/or extrahepatic metastasis, were found in 72.5% (100/138) of the patients. The diameter of the tumor in the Group of hepatitis B core antibody-positive [HBcAb(+)] and hepatitis B surface antibody-positive [HBsAb(+)] was 7.03 ± 3.76 cm, which was much smaller than 8.79 ± 4.96 cm in the Group of HBcAb(+) and HBsAb(−) (*P* = 0.035).

**Conclusion:**

It is estimated that at least 21 million OBI patients live in China. HBcAb(+) was not only the evidence of chronic HBV infection but also a dangerous mark for surface antigen-negative patients. A semi-annual or annual medical checkup is essential for all OBI patients to identify HCC as early as possible. The hypothesis underlying our analysis was that hepatitis B surface antibody would prevent the progress of HCC and facilitate the clearance of HBV in patients with OBI. Thereby, the hepatitis B vaccine could be used to prevent severe disease consequences.

## Introduction

According to the latest WHO report, 257 million individuals have HBsAg-positive chronic HBV infections. The Western Pacific Region has the greatest frequency of hepatitis B infection, with 6.2% of the adult population afflicted. Somewhere in the region of 20–30% of adults with HBV chronic infection will develop cirrhosis and/or liver cancer.

According to recent reports (Fu and Chen, 2020), there are more than 800,000 new cases and deaths of hepatocellular carcinoma each year. Chinese patients account for more than 50% of these. There are at least 86 million HBsAg-positive chronic HBV infections in China, but many OBI patients are still unknown.

For Chinese patients, chronic HBV infection is the most common cause of HCC. HBsAg positivity is often used as a marker for HBV infection, but this discriminant method is insufficient. When HBsAg negative, but HBcAb positive, with or without being hepatitis B e antibody (HBeAb) positive and/or HBsAb positive, this may be a manifestation of acute HBV infection in the months following. It also is found in chronic HBV infection. HBsAg turning negative does not mean HBV DNA clearance, and many of these patients may have OBI(Raimondo et al., 2019). Among patients with OBI, HBV may replicate at low levels for a long time with detectable or undetectable HBV DNA in the serum and liver (Raimondo et al., 2013). Wong DK reported an analysis of clinical data and liver tissues from 90 HBsAg negative patients with HCC in Chinese Hong Kong (Wong et al., 2019). They found that almost 70% had chronic OBI, of whom 70% integrated HBV DNA into liver cell DNA; 90% of these patients did not have cirrhosis. HBV DNA integrated near hepatic oncogenes; these integrations might promote the development of liver cancer.

Unfortunately, there is no detailed report from mainland China about the prevalence of N-HBsAg chronic HBV infection and its role in HCC patients. In recent years, we have performed a retrospective analysis about HCC patients related to N-HBsAg chronic HBV infection treated in our hospital.

## Materials and Methods

### Trial Design

We conducted the trial at The First Affiliated Hospital of Chongqing Medical University. The trial was conducted following Good Clinical Practice guidelines and the Declaration of Helsinki. All patients were patients diagnosed and treated in the hospital. All the authors designed the trial, collected the data, performed the analysis, and wrote the manuscript.

### Patients

Patients were hospitalized and diagnosed as HCC associated with HBV infection from May 2016 through May 2019 in The First Affiliated Hospital of Chongqing Medical University. The clinical diagnostic criteria refer to primary liver cancer diagnosis and treatment code 2019 edition (National Health and Health Commission of the people’s Republic of China, 2019).

Patient inclusion criteria: (1) HBsAg negative and HBeAg negative but HBcAb positive with or without HBeAb, HBsAb positive; (2) HCC was first diagnosed pathologically after percutaneous biopsy or surgical resection; (3) If without biopsy or surgery, there were at least two kinds of imaging tests of computerized tomography (CT), magnetic resonance imaging (MRI), and ultrasound contrast enhancement showed “fast in and fast out” imaging manifestations of typical HCC.

Patients exclusion criteria: (1) CT or MRI scan suspected metastatic tumor, and the primary lesion was unknown; (2) Hepatobiliary cell carcinoma was considered in CT or MRI; (3) Liver benign tumor, liver abscess, liver tuberculosis, and other infectious lesions could not be identified in CT or MRI.

The patients were classified into Early Group (stage 0 and A) and Advanced Group (stage B and C and D) by BCLC score (Easl Clinical Practice., 2018) shown in Table 1.

**TABLE 1:** HCC stage grouping criteria.

Reference	Early group (stage 0 and A)	Advanced group (stage B and C or D)
EASL clinical practice guidelines: management of hepatocellular carcinoma	Very early stage (0): single <2 cm Preserved liver function 1, PS 0	Intermediate stage(B): multinodular, unresectable Preserved liver function 1, PS 0
	Early-stage (A): single or 2–3 nodules <3 cm Preserved liver function 1, PS 0	Advanced stage (C): portal invasion/extrahepatic spread Preserved liver function 1, PS 1–2 Terminal stage (D) Not transplantable HCC End-stage liver function PS 3–4
		

### Analytical Data

Epidemiological and clinical data were analyzed, including age, sex, residence, initial symptoms, history of HBV or/and hepatitis C virus (HCV) infection, history of other hepatitis, history of cancer, history of diabetes, history of drinking, history of smoking and family history of hepatitis B.

Laboratory examination included serological data, such as alpha-fetoprotein (AFP, Upper Limit of Normal was 25.0 ng/ml), alanine aminotransferase (ALT, Upper Limit of Normal was 40 U/L), aspartic acid aminotransferase (AST, Upper Limit of Normal was 35 U/L), gamma-glutamyl transpeptidase (GGT, Upper Limit of Normal was 45 U/L) and total bilirubin (TB, Upper Limit of Normal was 21 μM/L).

Virological data included HBV (HBsAg, HBsAb, HBeAg, HBeAb, HBcAb, and HBV DNA) and HCV (anti-HCV IgG, HCV RNA).

Pathological examination of liver lesions was carried out by Liver biopsy or/and hepatectomy.

Imaging tests including CT, MRI, and ultrasound were applied to confirm tumor size and whether intrahepatic or extrahepatic metastasis had occurred.

Treatment options of the patients included hepatectomy surgery, transhepatic arterial chemotherapy and embolization (TACE), conservative treatment (none surgery and none chemotherapy), and abandoned treatment.

### Statistical Analysis

The statistical analysis and data management plans are included with the protocol at NEJM.org. With a total of 138 patients, we detected such a difference using the Chi-square test, *t*-test, assuming two-sided tests and a significance level of 0.05.

## Results

### Patients

**FIGURE 1:**
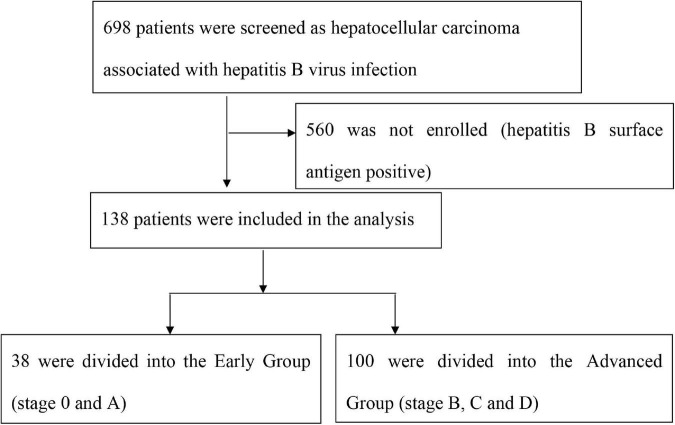
The HCC stage grouping criteria.

Among 698 patients screened as HCC associated with chronic HBV infection, a total of 138 patients were enrolled as N-HBsAg occult hepatitis B infections and were divided into Early Group and Advanced Group, see Figure 1.

The Comparison of some characteristics of early and advanced hepatocellular carcinoma is shown in Table 2.

**TABLE 2:** Comparison of some characteristics of early and advanced hepatocellular.

Items	All patients (*N* = 138)	Early group (*N* = 38)	Advanced group (*N* = 100)	*P*-value
N° patients n° (%)	138	38 (27.5)	100(72.5)	
Mean age (±SD)	63.2(10.8)	63.6(9.8)	63.0(11.2)	0.77*
Males n° (%)	110(79.7)	30(76.3)	80(80.0)	0.89**
Rural residents n° (%)	61(44.2)	18(47.4)	43(43.0)	0.64**
History of drinking n° (%)	66(47.8)	19(50.0)	47(47.0)	0.75**
History of smoking n° (%)	77(55.8)	23(60.5)	54(55.0)	0.49**
Patients with diabetes n° (%)	17(12.3)	4(10.5)	13(13.0)	0.69**
Anti-HCV-positive and HCV-RNA-positive n° (%)	5(3.6)	1(3.0)	4(4.0)	0.59**
Lesion found on physical examination n° (%)	30(21.7)	12(31.6)	18(18.0)	0.08**
History of HBV infection n° (%)	15(10.9)	3(7.9)	12(12.0)	0.49**
HBcAb(+) n° (%)	17 (12.3)	2 (5.3)	15(15.0)	0.21**
HBcAb(+), HBeAb(+) n° (%)	35 (25.4)	10 (26.3)	25(25.0)	0.87**
HBcAb(+), HBsAb(+), HBeAb(+) n° (%)	53 (38.4)	16 (42.1)	37(37.0)	0.58**
AST/ULN(mean ± SD)	3.34 (5.29)	2.36 (3.34)	3.71(5.84)	0.18*
ALT/ULN(mean ± SD)	1.91 (2.63)	1.96 (3.03)	1.89(2.48)	0.09*
GGT/ULN(mean ± SD)	6.40 (5.92)	4.65 (5.71)	7.07(5.88)	0.03*
TB, umol/L (mean ± SD)	45.8 (90.4)	27.48 (27.20)	52.07(105.15)	0.03*
AFP,ng/ml, (mean ± SD)	15564.96(79392.83)	551.85(3240.93)	21412.97(74762.43)	0.006*
HBV DNA positive n° (%)	3 (2.2)	2 (5.3)	1(1.0)	0.89**
HCV- IgG positive n° (%)	5 (3.6)	1(2.6)	4 (4.0)	0.59**

*t-test, **the chi-square test.

In the 138 HCC patients associated with OBI, 110 patients (79.7%) were men, and 28 patients (20.3%) werewomen. The men in the Early Group and the Advanced Group were 78.9% (30/38)and 80.0% (80/100), respectively. There was no statistical difference between them (*P* = 0.89). The median age was 63.2 years. The youngest patient was 38 years of age, and the oldest was 91 years of age. The median age of the Early Group and the Advanced Group was 63.6 and 63.0; respectively, there was no statistical difference between them (*P* = 0.77). In the overall trial population, 44.2% of patients were rural residents, 55.8% were urban residents. The percentage of urban residents in the Early Group and the Advanced Group was 52.6% (20/38) and 57% (57/100), respectively, and there was no statistical difference between them (*P* = 0.64).

Before being hospitalized, most of the trial population (108/138, 78.3%) complained of initial symptoms including right upper abdominal discomfort or/and pain (63/138, 45.7%), poor appetite, abdominal distension, and other gastrointestinal symptoms (45/138, 32.6%). Further, 36 patients (26.1%) had hepatosplenomegaly, 32 patients (23.2%) had portal hypertension, and 16 patients (11.6%) had ascites. Unfortunately, simple liver fibrosis and steatosis are rarely observed in imaging due to late detection or lack of liver elasticity and fat detection 30 patients (21.7%) had no symptoms and were found to have HCC by health examination, 12 patients belonged to the Early Group, and 18 patients belonged to the Advanced Group. There was no statistical difference between them (*P* = 0.08).

In the total 138 trial population, 15 patients (10.9%) had a history of chronic HBV infection, and only 3 patients (2.2%) had a family history of hepatitis B. Only 5 patients (3.6%) admitted had a history of chronic HCV infection, 6 patients (4.3%) a history of cancer, and none had any exposure to a hepatotoxic substance. A total of 17 patients (12.3%) had diabetes, the percentage of the Early Group and the Advanced Group was 10.5% (4/38) and 13.0% (13/100), respectively, there was no statistical difference between them (*P* = 0.69). It was found that 66 patients (47.8%) had a history of drinking, the percentage of the Early Group and the Advanced Group was 50.0% (19/38) and 47% (47/100), respectively, there was no statistical difference between them (*P* = 0.75). Furthermore, 77 patients (55.8%) had a history of smoking, the percentage of the Early Group and the Advanced Group was 60.5% (23/38) and 54.0% (54/100), respectively, there was no statistical difference between them (*P* = 0.49). Older men (79.7%), cigarette (55.8%), and alcohol (47.8%) consumers were the top three populations of HCC associated with OBI.

### Laboratory Test Results

The Laboratory Test Results/Imaging Findings and Treatment options of the Patients are shown in Table 3.

**TABLE 3:** Comparison of baseline and HCC characteristics between the HBsAb Positive Group and the HBsAb Negative Group.

Items	HBsAb positive group	HBsAb negative group	*P*-value
N° patients n° (%)	86(62.3)	52(37.7)	
Mean age (± SD)	63.5(11.5)	62.8(9.6)	0.72*
Males n° (%)	67(77.9)	43(82.7)	0.50**
History of drinking n° (%)	39(45.3)	27(51.9)	0.45**
History of smoking n° (%)	48(55.8)	29(55.8)	1.00**
Patients with diabetes n° (%)	8(9.3)	9(17.3)	0.17**
Anti-HCV-positive and HCV-RNA-positive n° (%)	1(1.2)	4(7.7)	0.06**
Lesion found on physical examination n° (%)	18(20.9)	12(23.1)	0.77**
History of HBV infection n° (%)	7(8.1)	8(15.4)	0.19**
AST/ULN(mean ± SD)	3.40(6.24)	3.23(3.22)	0.86*
ALT/ULN(mean ± SD)	1.83(2.49)	2.03(2.87)	0.67*
GGT/ULN(mean ± SD)	5.96(5.78)	7.13(6.12)	0.27*
TB, umol/L (mean ± SD)	49.76(108.18)	37.92(52.29)	0.46*
AFP,ng/ml (mean ± SD)	19825.99(73972.37)	8792.91(43536.67)	0.33*
112(81.2%) cases with the maximum diameter of the largest tumor lesion (cm mean ± SD)	7.03(3.76)	8.79(4.96)	0.035*

*t-test, **the chi-square test.

#### Liver Function

The positive rates of ALT and AST were 60.9% (84/138) and 76.8% (106/138), respectively, the mean values were no statistical difference between the Early Group and the Advanced Group (*P* = 0.09, *P* = 0.18, respectively). The positive rate of GGT was 86.2% (119/138), the mean values of GGT/ULN in the Early Group and the Advanced Group were 4.65 ± 5.71, and 7.07 ± 5.88, respectively, the difference between them was statistically significant (*P* = 0.03), which showed that GGT in the Advanced Group increased more obviously than that in the Early Group. The positive rate of TB was 47.8% (66/138), the mean values of TB in the Early Group and the Advanced Group were 27.48 ± 27.20 and 52.07 ± 105.15, respectively, TB in the Advanced Group increased more obviously than that in the Early Group, the difference between the two groups was statistically significant (*P* = 0.03).

#### Serological Molecular Marker Diagnosis for Hepatocellular Carcinoma

AFP was tested positive in 54 patients (39.1%). The mean values of AFP in the Early Group and the Advanced Group were 551.85 ± 3240.93 ug/L and 21412.97 ± 74762.43 μg/L, respectively, which in the Advanced Group was much higher than that in the Early Group (*P* = 0.006).

#### The Virological Characteristics of Hepatitis B and C Viruses

All 138 patients were tested for hepatitis B and C virus markers in serum. HBcAb was found positive in all 138 patients. Both of HBcAb and HBsAb were positive in 33 patients, and the positive rates in the Early Group and the Advanced Group were 28.9% (11/38) and 22% (22/100), respectively, there was no statistical difference between the two groups (*P* = 0.39). The serum concentration of HBsAb was 10.4 to 1000 mIU/ml, and its average value was 179.9 mIU/ml.

Both of HBcAb and HBeAb were found positive in 35 patients, and the positive rates of these two antibodies in the Early Group and the Advanced Group were 26.3% (10/38) and 25.0% (25/100), respectively, there was no statistical difference between them (*P* = 0.87).

HBcAb, HBsAb, and HBeAb positive results were found in 53 patients, and the positive rates of the Early Group and the Advanced Group were 42.1% (16/38) and 37.0% (37/100), respectively, there was no statistical difference between them (*P* = 0.58). The serum concentration of HBsAb was 10.47 to 1000 mIU/ml, and its average value was 209.1 mIU/ml.

HBcAb positive solely was found in 17 patients (12.3%), the positive rates of the Early Group and the Advanced Group were 5.3% (2/38) and 15.0% (15/100), respectively, there was no statistical difference between them (*P* = 0.21).

There was no HBcAb negative, and HBsAb and HBeAb positive patients were found in our trial.

Serum HBV DNA was found positive in three patients (2.2%), their positive markers of hepatitis B virus are shown below:

Case1, HBsAb 68.48mIU/ml(+), HBeAb 0.03 S/CO(+), HBcAb 5.42 S/CO(+), HBV DNA 3.20 × 10^3^ IU/ml.

Case2, HBsAb 178.04mIU/ml(+), HBeAb 0.01S/CO(+), HBcAb 10.79 S/CO(+), HBV DNA 5.95 × 10^4^ IU/ml.

Case 3, HBsAb 61.20mIU/ml(+), HBeAb(−), HBcAb 3.32S/CO(+), HBV DNA 1.48 × 10^6^ IU/ml.

Hepatitis C virus (HCV) antibody IgG (HCV-IgG) positive was found in 5 patients (5/138, 3.6%), 1 (1/38, 3.0%) in the early Group and 4 in the Advanced Group (4/100, 4.0%), there was no statistical difference between them (*P* = 0.59).

HCV-IgG and HCV RNA positive were found in three patients (2.2%), their serum markers of HBV and HCV are shown below:

Case 1, HBsAb (−), HBeAb (−), HBcAb 6.15s/co (+). HCV-IgG (+), HCV RNA 1.32 × 10^4^ IU/ml.

Case 2, HBsAb (−), HBeAb (−), HBcAb 2.81s/co (+). HCV-IgG (+), HCV RNA 1.48 × 10^6^ IU/ml.

Case 3, HBsAb 61.20mIU/ml (+), HBeAb (-), HBcAb 3.32s/co (+). HCV-IgG (+), HCV RNA 1.60 × 10^5^ IU/ml.

### Imaging Findings and Treatment Options

All 138 patients had CT and MRI scans, shown in Table 3. The number of patients with the imaging examination stage 0 and A, stage B, stage C, and stage D were 38 (27.5%), 25 (18.1%), 63 (45.7%), and 12 (8.7%), respectively. There were 109 patients (78.9%) without vascular invasion and 29 patients (21.1%) with. In total 57 patients (41.3%) were found intrahepatic metastasis, and 25 patients (18.1%) had extrahepatic metastasis, and all of them belonged to the Advanced Group. Only one patient also recieved 18F-FDG PET/CT examination, which revealed HCC with intrahepatic, double lung, bone, and mediastinal metastases. Meanwhile, 23 patients (16.7%) also received liver biopsy and were diagnosed as having HCC with cirrhosis.

Regarding the treatment options of the patients, unfortunately only 19.6% (27/138) of patients were suitable for surgical resection of HCC by hepatic lobectomy, 21.0% (29/138) of patients chose transhepatic arterial chemotherapy and embolization (TACE), the remaining 59.4% (82/138) of patients had no chance of surgery or TACE and chose conservative treatment or refused any treatment.

### Statistical Analysis

A comparative study is shown in Table 2, between the Early Group and the Advanced Group there was no significant difference in age, gender, residence, history of HBV infection, history of drinking and smoking, number of HCV overlapping infection, number of diabetes, initial symptoms, ALT and AST values, HBsAb positive rate, HBeAb positive rate.

**FIGURE 2:**
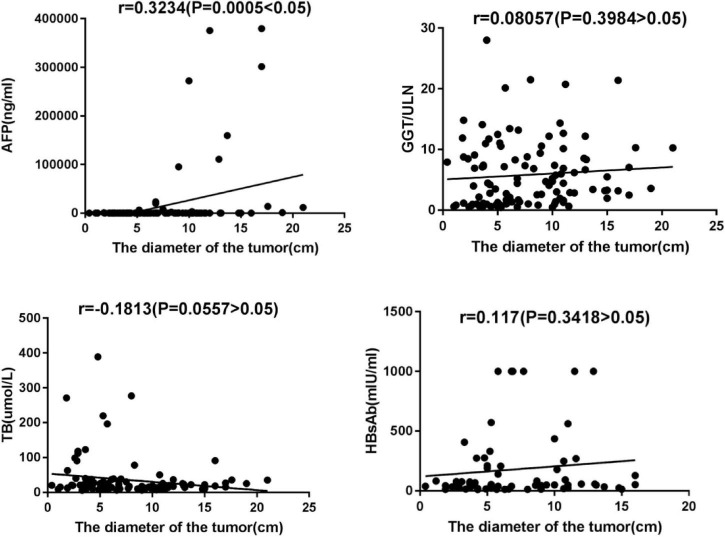
Correlation between AFP/GGT/TB and HBsAb value and maximum tumor diameter.

However, GGT, TB, and AFP values in the Advanced Group were significantly higher than those in the Early Group. The value of AFP was positively correlated with tumor diameter (*r* = 0.3234, *P* = 0.005). There was no significant correlation between GGT, TB, and tumor diameter, see Figure 2.

In our study, HBcAb seropositive was found in all 138 patients (100%), HBsAb seropositive was in 86 patients (62.3%). According to HBsAb positivity, 138 patients were divided into HBsAb Positive Group and HBsAb Negative Group and their baseline characteristics comparatively analyzed, and there was no statistically significant difference between them as shown in Table 2. The diameter of the largest tumor lesion was accurately measured in 112 patients (81.2%), and comparative analysis revealed that the mean maximum tumor diameter of the HBsAb Positive Group was significantly smaller than that of the HBsAb Negative Group (*P* = 0.035). Furthermore, the correlations between the serum value of HBsAb and maximum tumor diameter in the HBsAb Positive Group were analyzed, but the result was not statistically significant (see Figure 2).

## Discussion

Our data indicated that occult hepatitis B infection patients were substantial and should be treated seriously. According to findings from this study, OBI-related HCC accounted for about 19.8% of the HBV-associated HCC. It is estimated that at least 21 million OBI patients live in China, and 86 million hepatitis B surface antigen-positive patients have chronic HBV infections. Unfortunately, most of the general public and medical workers alike pay no attention to up-to-date OBI patients who are still considered healthy and are paid no attention.

Our data indicated that older men (79.7%), cigarette (55.8%), and alcohol (47.8%) consumers were the top three high-risk population of HCC associated with OBI. This was consistent with the reports of other researchers worldwide (Yuen et al., 2008; Lyu et al., 2016; Yip et al., 2017; Baecker and Zhang, 2018). Unfortunately, the risk factors of hepatic steatosis and hepatic fibrosis for HCC were not observed. Therefore, it is essential for OBI patients to quit drinking and smoking, especially young men.

Our data indicated that most of the patients did not have a clear history of chronic HBV infection or family history of HBV infection, and they were not tested for HBV or HCV and did not know HBV had infected them until having obvious symptoms and being hospitalized. Therefore, it is essential to take a semi-annual or annual medical check, including serum HBV, HCV tests, liver CT, or MRI scanning for all OBI patients, especially in middle aged and older men.

Our data indicated that AFP serologically positive was inadequate for the diagnosis of HCC, although it is still important to monitor the occurrence and prognosis of HCC in patients with chronic hepatitis B and HCC differentiation, size, and vascular invasion have strong relationships with AFP, The AFP level at diagnosis was an independent risk predictor associated with pathological grade, progression, and survival(Xu et al., 2012; Liu et al., 2013; Chun et al., 2015; Meng et al., 2016; Yao and Lu, 2016; Bai et al., 2017; Yang et al., 2018). On the contrary, CT and/or MRI scan imaging plays a notable role in HCC surveillance, diagnosis, and treatment response assessment, consistent with other reports (Vietti Violi et al., 2019; Singal, 2020).

Our results confirmed that hepatitis B core antibody-positive was the evidence of HBV infection and a dangerous mark for all OBI patients, similar to other reports (Cacciola et al., 1999; Fattovich et al., 2008; Matsue et al., 2010; Coppola et al., 2014, 2016; Lee et al., 2015; Law et al., 2019; Qu et al., 2019; Suzuki et al., 2019). Furthermore, our findings showed that the mean maximum tumor diameter of the HBsAb Seropositivity Group was significantly smaller than that of the HBsAb Seronegative Group. The hypothesis underlying our analysis was that hepatitis B surface antibody would interfere with the progress of HCC and facilitate the clearance of HBV in patients with OBI, thereby hepatitis B vaccine could be used to prevent the most severe disease consequences.

## Author Contributions

YY, RX, JP, JH, PJ, CY, and XL initiated the study and participated in data collection. RX summarized and analyzed the data and then wrote a manuscript. YY made the final revision to the manuscript. All authors contributed to the article and approved the submitted version.

## Conflict of Interest

The authors declare that the research was conducted in the absence of any commercial or financial relationships that could be construed as a potential conflict of interest.

## Publisher’s Note

All claims expressed in this article are solely those of the authors and do not necessarily represent those of their affiliated organizations, or those of the publisher, the editors and the reviewers. Any product that may be evaluated in this article, or claim that may be made by its manufacturer, is not guaranteed or endorsed by the publisher.
