# Supplementary material for: The Serious Challenge of Occult Hepatitis B Virus Infection-Related Hepatocellular Carcinoma in China
Source: Front Microbiol. 2022 Feb 7;13:840825. doi: 10.3389/fmicb.2022.840825 (PMC8859271; doi:10.3389/fmicb.2022.840825)
Supplement: Supplementary file 1 [file Data_Sheet_1.docx]

Table 1：HCC Stage Grouping Criteria

| Reference | Early Group (stage 0 and A) | Advanced Group (stage B and C or D) |
| --- | --- | --- |
| EASL Clinical Practice Guidelines: Management of hepatocellular carcinoma | Very early stage (0)：Single <2 cm  Preserved liver function 1,  PS 0 | Intermediate stage(B): Multinodular, unresectable  Preserved liver function 1,  PS 0 |
|  | Early-stage (A): Single or 2-3 nodules <3 cm  Preserved liver function 1,  PS 0 | Advanced stage (C): Portal invasion/extrahepatic spread  Preserved liver function 1,  PS 1 -2  Terminal stage (D)  Not transplantable HCC  End-stage liver function  PS 3-4 |

Table 2：Comparison of some characteristics of early and advanced hepatocellular carcinoma

| Items | All Patients (N=138) | Early Group  (N=38) | Advanced Group  (N=100) | P value |
| --- | --- | --- | --- | --- |
| N° patients n° (%) | 138 | 38 (27.5) | 100(72.5) |  |
| Mean age (±SD) | 63.2(10.8) | 63.6(9.8) | 63.0(11.2) | 0.77* |
| Males n° (%) | 110(79.7) | 30(76.3) | 80(80.0) | 0.89** |
| Rural residents n° (%) | 61(44.2) | 18(47.4) | 43(43.0) | 0.64** |
| History of drinking n° (%) | 66(47.8) | 19(50.0) | 47(47.0) | 0.75** |
| History of smoking n° (%) | 77(55.8) | 23(60.5) | 54(55.0) | 0.49** |
| Patients with diabetes n° (%) | 17(12.3) | 4(10.5) | 13(13.0) | 0.69** |
| Anti-HCV-positive and HCV-RNA-positive n° (%) | 5(3.6) | 1(3.0) | 4(4.0) | 0.59** |
| Lesion found on physical examination n° (%) | 30(21.7) | 12(31.6) | 18(18.0) | 0.08** |
| History of HBV infection n° (%) | 15(10.9) | 3(7.9) | 12(12.0) | 0.49** |
| HBcAb(+) n° (%) | 17 (12.3) | 2 (5.3) | 15(15.0) | 0.21** |
| HBcAb(+), HBeAb(+) n° (%) | 35 (25.4) | 10 (26.3) | 25(25.0) | 0.87** |
| HBcAb(+), HBsAb(+), HBeAb(+) n° (%) | 53 (38.4) | 16 (42.1) | 37(37.0) | 0.58** |
| AST/ULN(mean ± SD) | 3.34 (5.29) | 2.36 (3.34) | 3.71(5.84) | 0.18* |
| ALT/ULN(mean ± SD) | 1.91 (2.63) | 1.96 (3.03) | 1.89(2.48) | 0.09* |
| GGT/ULN(mean ± SD) | 6.40 (5.92) | 4.65 (5.71) | 7.07(5.88) | 0.03* |
| TB, umol/L (mean ± SD) | 45.8 (90.4) | 27.48 (27.20) | 52.07(105.15) | 0.03* |
| AFP,ng/ml, (mean ± SD) | 15564.96(79392.83) | 551.85(3240.93) | 21412.97(74762.43) | 0.006* |
| HBV DNA positive n° (%) | 3 (2.2) | 2 (5.3) | 1(1.0) | 0.89** |
| HCV- IgG positive n° (%) | 5 (3.6) | 1(2.6) | 4 (4.0) | 0.59** |

- *t*-test; ** the chi-square test.

Table3：Comparison of baseline and HCC characteristics between the HBsAb Positive Group and the HBsAb Negative Group

| items | HBsAb Positive Group | HBsAb Negative Group | P  value |
| --- | --- | --- | --- |
| N° patients n° (%) | 86(62.3) | 52(37.7) |  |
| Mean age (±SD) | 63.5(11.5) | 62.8(9.6) | 0.72* |
| Males n° (%) | 67(77.9) | 43(82.7) | 0.50** |
| History of drinking n° (%) | 39(45.3) | 27(51.9) | 0.45** |
| History of smoking n° (%) | 48(55.8) | 29(55.8) | 1.00** |
| Patients with diabetes n° (%) | 8(9.3) | 9(17.3) | 0.17** |
| Anti-HCV-positive and HCV-RNA-positive n° (%) | 1(1.2) | 4(7.7) | 0.06** |
| Lesion found on physical examination n° (%) | 18(20.9) | 12(23.1) | 0.77** |
| History of HBV infection n° (%) | 7(8.1) | 8(15.4) | 0.19** |
| AST/ULN(mean ± SD) | 3.40(6.24) | 3.23(3.22) | 0.86* |
| ALT/ULN(mean ± SD) | 1.83(2.49) | 2.03(2.87) | 0.67* |
| GGT/ULN(mean ± SD) | 5.96(5.78) | 7.13(6.12) | 0.27* |
| TB, umol/L (mean ± SD) | 49.76(108.18) | 37.92(52.29) | 0.46* |
| AFP,ng/ml (mean ± SD) | 19825.99(73972.37) | 8792.91(43536.67) | 0.33* |
| 112(81.2%) cases with the maximum diameter of the largest tumor lesion (cm mean ± SD) | 7.03(3.76) | 8.79(4.96) | 0.035* |

**t*-test，**the chi-square test.

698 patients were screened as hepatocellular carcinoma

associated with hepatitis B virus infection

560 was not enrolled (hepatitis B surface antigen positive)

138 patients were included in the analysis

38 were divided into the Early Group (stage 0 and A)

100 were divided into the Advanced Group (stage B, C and D)

Figure 1: The HCC stage grouping criteria.


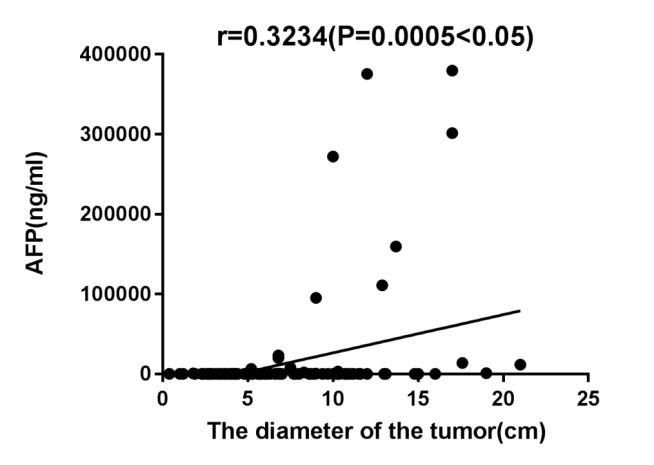

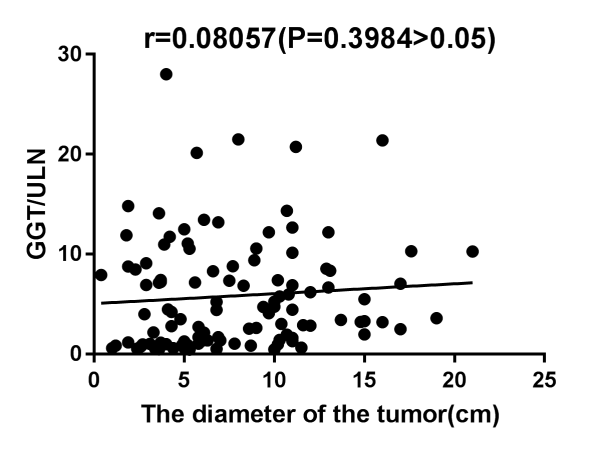

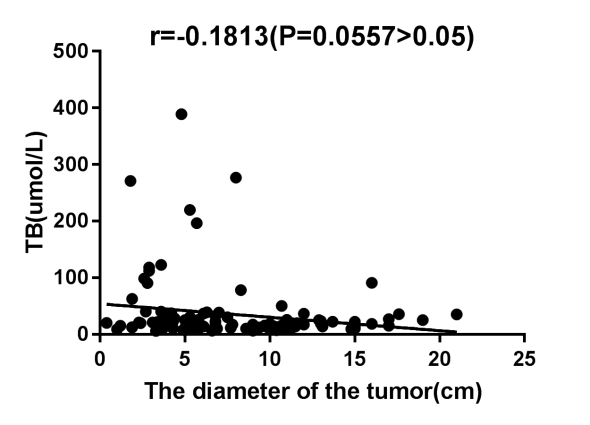

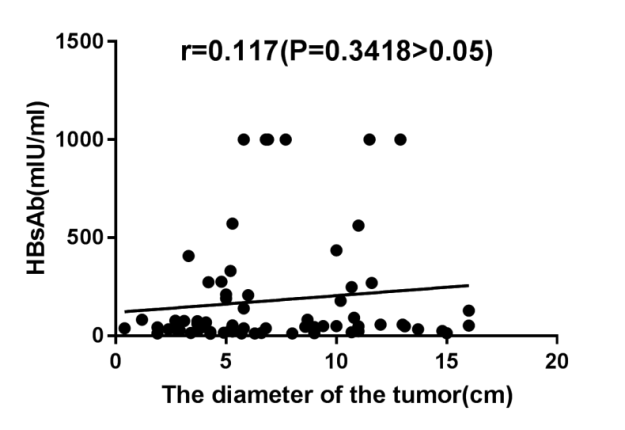


Figure 2: Correlation between AFP /GGT/TB and HBsAb value and maximum tumor diameter
